# Influence mechanism and impacting boundary of workplace isolation on the employee's negative behaviors

**DOI:** 10.3389/fpubh.2023.1077739

**Published:** 2023-03-09

**Authors:** Ya-Juan Yang, Lei Lu

**Affiliations:** ^1^City College of Dongguan, Dongguan, Guangdong, China; ^2^School of Business, Macau University of Science and Technology, Taipa, Macao SAR, China

**Keywords:** workplace isolation, organizational identification, identification orientation, work fatigue, turnover intention (TI)

## Abstract

**Introduction:**

Based on social identity theory, by introducing organizational identification as mediating variable and identification orientation as moderating variable, this paper studies the influence mechanism and impacting boundary of workplace isolation on employee fatigue and turnover intention.

**Methods:**

Based on logic relationship, seven basic hypotheses are put forward to construct the theoretical model of the problem. Based on the 300 effective questionnaires being obtained from employees in Mainland China, the empirical investigation adopts the three-phase lag time design. By regression analysis and bootstrap test.

**Results:**

(1) Workplace isolation has a significant positive effect on employee's work fatigue; (2) Workplace isolation has a significant positive effect on employee's turnover intention; (3) Organizational identification plays a partial mediating role between workplace isolation and work fatigue; (4) Organizational identification plays a partial mediating role between workplace isolation and employee's turnover intention; (5) Employee identification orientation negatively moderates the relationship between workplace isolation and organizational identification, that is to say, the higher the degree of identification orientation, the more inhibited the negative impact of workplace isolation on organizational identification; (6) Employee identification orientation has a negative moderating effect, namely, compared with the low degree of employee identification orientation, the higher the employee identification orientation, the positive effect of workplace isolation on work fatigue and turnover intention through organizational identification become weaker.

**Discussion:**

Understanding these influencing mechanisms will have a great influence on guiding managers to mitigate the negative effects of “workplace isolation” in practice and improve the work efficiency of employees.

## 1. Introduction

The global spread and normalization of COVID-19 have had a huge impact on people's work and life (1, 2), and “isolation” has also frequently appeared in different parts of society (3). In recent years, the management domain closely concerns another “isolation” belonging to the professional category of workplace form, which is quite different from the former “isolation.” With the rapid economic growth, the overall quality of workers in the whole society has improved while the competition in the workplace has increased and the interpersonal conflicts frequently occur. Especially, the “workplace isolation” became common and leads to increasingly serious management and social problems (4). In the United States, a survey of 262 employees in the workplace showed that 69% of employees believed that they were excluded and isolated by their leaders or colleagues during work (5); In China, a survey lasting 1 month conducted by a recruitment company on more than 10,000 current employees showed that nearly half of the employees believed that they had been isolated and excluded at work (6).

Under the Chinese organizational culture, workplace isolation is more reflected in cold violence due to the deep influence of traditional cultural concepts such as authority worship and the implicit and introverted life belief (6). Besides, workplace destructive behaviors are more in the form of implicit and indirect mental abuse (7), such as workplace gossip (8), and workplace isolation (7). In foreign workplaces, workplace isolation is more reflected in fierce violence based on the universal value of facing directly (9). Most destructive behaviors in the workplace involve direct physical conflicts (7), such as workplace aggression (10), workplace victimization (11).

The study of workplace destructive behavior in China should start from the cold violence in the workplace, such as exclusion and isolation. In the past, studies on workplace cold violence mostly focused on workplace exclusion (12–16). As a more hidden conflict than workplace exclusion, there are less researches on workplace isolation (4). Only a few papers involve the impact of workplace isolation on employees' active production behavior, such as lowering job performance (7, 12), reducing job satisfaction (7), suppressing employees' innovative behavior and narrowing teamwork (4).

Workplace isolation refers to the psychological construct including organizational isolation and colleague isolation perceived by employees, which lack of support from colleagues and superiors, and lack of social and emotional interaction opportunities with team members (7). In short, workplace isolation refers to employees' psychological perception of their organization, which is formed due to the lack of support from colleagues and leaders for work, as well as the lack of social and emotional interaction with team members. In addition, the overall impact of workplace isolation on employees' negative behavior has not been studied so far. As we all know, employees' negative behavior will bring further destructive effects to the organization, such as reducing the morale of organization members and affecting the development of the organization. The direct impact of employees' negative behavior comes from two aspects, namely work fatigue and turnover intention. Firstly, if employees feel extreme fatigue, their functional ability will be diminished. The negative effects are reflected in both the reduction of individual work efficiency and job satisfaction (14), and lead to negative or deferred response to work (17). Secondly, when employees are dissatisfied or feel frustrated in expectations, they will have the idea of leaving the organization (18), which will lead to the brain drain of the organization, lower the positivity of the organization, and damage the performance of the organization (19).

To sum up, this paper uses work fatigue and turnover intention as indicators to measure employees' negative behavior, and uses social identity theory as a guide to explore the mechanism of the negative effects exerted by workplace isolation. As mentioned above, this paper is intended to supplement the theory that is relatively neglected in this research field.

## 2. Theoretical framework

According to social identity theory, individuals derive their self-image from the social category they belong to Tajfel and Turner (20, 21). Therefore, employees will feel that they are not accepted by the organization because of workplace isolation, so they cannot integrate themselves into the organization, and it is difficult for them to become a member of the organization, which will reduce the identification of the organization. Naturally, if employees have to work when they have a low sense of organizational identification and can't be accepted by the organization, it will result in work fatigue (22), which will trigger employees' turnover intention for a long time. In addition, individual behavior is determined by the organizational environment where they are located and their own internal characteristics (23). So, employees' negative behaviors, such as work fatigue and even resignation, are affected by the organizational environment and their own characteristics. On the one hand, employees are affected by work isolation. This unaccepted and excluded organizational environment affects employees' positivity to integrate emotionally, which leads to negative work behaviors. On the other hand, employees' own characteristics will also affect their work behavior. According to the self-identity orientation theory (24), employees' identification orientation is an individual preferred personality trait defined by themself, which will affect the perception and interpretation of external information (25). Employees' identification orientation is characterized by meeting personal needs and achieving self-improvement (26). Although workplace isolation makes it difficult for employees to integrate into the organization at the level of organizational environment, employees' self-defined preferred identification orientation can help employees generate recognition of the organization with their own characteristics and inhibit the occurrence of work fatigue and turnover intention.

In view of this, we explore the impact mechanism of workplace isolation on employees' negative behavior from the dual perspective of organizational environment and individual characteristics, and focus on three following issues. (1) the impact of workplace isolation on employees' work fatigue and turnover intention from the aspect of organizational environment. (2) the mediating effect of organizational identification when workplace isolation affects work fatigue and turnover intention based on social identity theory. (3) the moderating role and impact boundary of employee identification orientation from the perspective of individual characteristics.

## 3. Theoretical hypotheses

### 3.1. Workplace isolation and work fatigue

The characteristics of workplace isolation are as follows. Firstly, when employees are working, they need to invest more resources to complete task. But they feel tired because they are isolated by the organization and colleagues. Secondly, the essence of workplace isolation stems from the lack of emotion and opportunity between colleagues (7). Without communication objects, employees lack emotional communication and catharsis leads to employees' depression. At the same time, employees feel helpless and exhausted in the face of work tasks (27); Thirdly, employees isolated by the organization cannot access core tasks, core personnel and core technologies (4). Due to the inability to find core personnel and learn core technologies, their ability to solve problems are limited, and they often feel helpless and hopeless, which then leads to work fatigue. Fourthly, workplace isolation usually leads to unfair treatment, such as unequal development opportunities, remuneration and promotion opportunities. Even if work harder and study more actively, isolated employees may still receive unequal treatment, which will make employees feel disappointed, generate negative thoughts, and increase their sense of work fatigue; Fifthly, for individuals, intimacy can predict individual health and wellbeing, while loneliness and isolation are more likely to make individuals experience health problems such as stress, anxiety and fatigue (28). Isolated employees are more likely to experience frustration, powerlessness, lack of opportunities for career development in the future, so they will lose work initiative, which result in work fatigue (29); Finally, isolation will infect and influence each other among employees, resulting in egative behavior of the organization team. Based on the above analyses, this paper proposes the first hypothesis:

***Hypothesis 1: workplace isolation has a significantly positive impact on employees' work fatigue***.

### 3.2. Workplace isolation and turnover intention

Turnover intention refers to the intention to leave the organization of employees for various reasons after working in the organization for a period of time (30). Firstly, due to the lack of cooperation with supervisors and colleagues, employees have to complete tasks without help and guidance, which leads to employees' turnover intention. Secondly, when encountering difficulties at work, employees suffering workplace isolation have no colleagues to communicate and can't get rid of their bad mood, which will inevitably lead to negative work attitude and turnover intention (31). Thirdly, workplace isolation will make employees fail to integrate into colleagues and teams, resulting in interpersonal alienation. The isolated employees will subconsciously keep distance from their colleagues or teams and lack the sense of belonging, resulting in the idea of resignation (32). Finally, workplace isolation accompanies unfair treatment. When employees feel treated unfairly, they will have the idea of leaving the organization (33). At the same time, isolated employees are unwilling or don't dare to express their ideas, and ignored during group decisions, which will also give rise to the leaving tendency of employees.

The isolation employees facing in the workplace are not only the subjective intentional isolation of their colleagues and leaders at work, but also the unintentional exclusion in the organization (4). On the one hand, this intentional and unintentional exclusion makes employees feel abandoned by the mainstream group psychologically. In order to vent their bad mood, employees may leave the organization. On the other hand, employees who affected by isolation are facing the risk of losing resources (positive emotions, self-efficiency). In order to avoid the loss of resources, employees will also have turnover intention. In view of this, this paper proposes the second hypothesis:

***Hypothesis 2: workplace isolation has a significantly positive impact on employee turnover intention***.

### 3.3. The mediating role of organizational identification

Organizational identification is a kind of perception belonging to the organization, which means that individuals define themselves as a member of the organization (34). According to the social identity theory, organizational identification is a special form of social identification. Individuals often need to obtain self-concept from the positioning of the relationship with the organization and others (6, 21). The most critical way to obtain self-concept for employees is the external identification (21). If employees are isolated by other members of the organization, they will feel that they are not accepted by the organization, which will reduce the organizational identification and distort their self-awareness.

Workplace isolation will inhibit employees' organizational identification, mainly because workplace isolation will pose a threat to people's basic needs. First, workplace isolation affects people's need to belongings. Human beings are certain kind of social animals who want to belong to certain groups or organizations and maintain certain social connections (35). Due to the lack of communication and emotional exchange, workplace isolation blocks the social contact between isolated employees and others, thus undermining people's need for belonging. Second, workplace isolation affects people's need to self-esteem. Self-esteem is an important factor for individuals to generate and maintain positive emotions (36). Workplace isolation makes isolated employees unwelcome due to the lack of support from colleagues and leaders. Because of being despised and neglected by others and organizations, isolated employees' positive emotions, like self-confidence, wellbeing and self-esteem, will be damaged. Third, workplace isolation affects people's need to control. People want to maintain a certain control over the surrounding to reduce the impact of environmental uncertainty on them (37). Workplace isolation affects the isolated employees' sense of control over interpersonal interaction. Isolated employees are difficult to get support and response from others and cannot meet their own control needs. Fourth, workplace isolation affects people's need to meaningful existence (6, 38). Workplace isolation deprives isolated employees of the meaningful existence within the organization. Because isolated employees normally have no access to core tasks and key leaders, they feel themself unnecessary and insignificant in the organization. Obviously, workplace isolation cannot meet employees' needs for belonging, self-esteem, control and existence, which will weaken employees' sense of identification with the organization. In addition, the decline of employees' organizational identification will lead to their negative emotions toward work and turnover intention (39).

If employees' organizational identification is threatened, employees' emotional connection to the organization will be destroyed, and employees' overall awareness of organizational relevance will be reduced (40, 41). In this working state, employees try to leave the organization and are unwilling to continue to work hard to achieve organizational goals. Even if they decide to stay in the organization, it is difficult for employees to devote themselves to work and easy to feel tired. On the contrary, if an employee has a high degree of identification with the organization, it means that he or she has the group consciousness and believes that he or she is an indispensable part of the organization (40). In this way, the employee will associate organizational achievements with personal achievements, so he or she is willing to put more efforts into it. To achieve organization's mission and long-term goals, he or she will spare no effort to work.

In short, due to workplace isolation, employees cannot get the support and guidance of colleagues or leaders at work, nor can they get emotional communication and interpersonal interaction. They feel it difficult to integrate into the organization and be accepted by the organization. Isolated employees are difficult to meet the needs of belonging, self-esteem, control and existence at work. Without the organizational identification, they feel tired at work and even try to leave the organization. According to these, another two hypotheses are proposed:

***Hypothesis 3: organizational identification plays a mediating role in the relationship between workplace isolation and work fatigue; Workplace isolation will inhibit employees' identification with the organization, resulting in employees' work fatigue***.

***Hypothesis 4: organizational identification plays a mediating role in the relationship between workplace isolation and turnover intention; Workplace isolation will inhibit employees' identification with the organization, resulting in employees' turnover intention***.

### 3.4. The moderating role of identification orientation

Self-identity orientation theory (24) believes that individuals have their own ways to obtain a sense of belonging in their environment, and personal identification orientation is a stable personality trait (25). Employees' identification orientation shows strong motivation for self-improvement (26). Although workplace isolation reduces employees' sense of identification with the organization, employees can alleviate the negative impact of workplace isolation on organizational identification through self-improvement and self-support personality traits.

For employees with high identification orientation, they can achieve self-improvement through proactive behavior, and then identify with the organization during work. As intrinsic motivation, self-improvement is an important driving factor to motivate employees to implement positive work behavior (25). This intrinsic motivation of self-improvement can inhibit or even offset the low organizational identification caused by workplace isolation. In addition, individual identification orientation can alleviate the negative results caused by organizational inequity (42). Workplace isolation actually originates from the unfair treatment of colleagues and leaders. This unfair treatment makes employees unable to integrate into the organization, which reduces employees' sense of identification with the organization. However, employees with high personal identification orientation, with the intrinsic motivation of self-improvement, are willing to seize the opportunity and prove their ability to curb the negative impact. Therefore, we propose the following hypothesis 5:

***Hypothesis 5: identification orientation negatively moderates the relationship between workplace isolation and organizational identification. Namely, the stronger the identification orientation of employees, the weaker the negative impact of workplace isolation on organizational identification and vice versa***.

### 3.5. The moderated mediating model

Combining the mediating effect of hypothesis 3 and 4 with the moderating effect of Hypothesis 5, a moderated mediating model is established. Organizational identification plays a mediating role not only between workplace isolation and work fatigue, but also between workplace isolation and turnover intention. However, these mediating effects are affected by identification orientation. Specifically, for employees with high identification orientation, the positive effect of workplace isolation on work fatigue and turnover intention through organizational identification will be weakened, which leads to hypothesis 6 and hypothesis 7.

***Hypothesis 6: identification orientation negatively moderates the positive and indirect relationship between workplace isolation and work fatigue through organizational identification. Namely, the stronger the identification orientation of employees, the weaker the positive indirect relationship and vice versa***.

***Hypothesis 7: identification orientation negatively moderates the positive and indirect relationship between workplace isolation and turnover intention through organizational identification. Namely, the stronger the identification orientation of employees, the weaker the positive indirect relationship and vice versa***.

**Figure 1:**
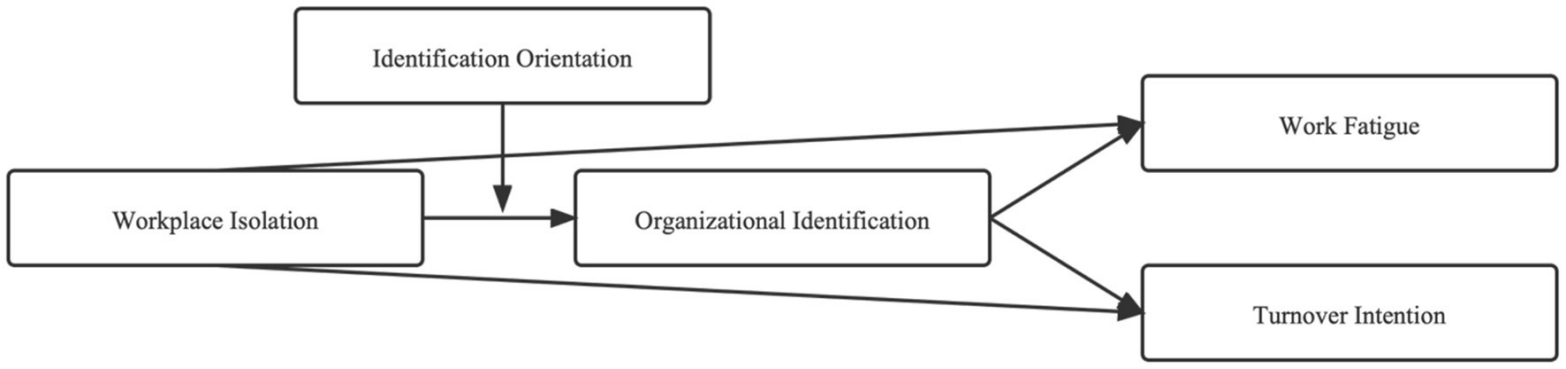
Research framework–theoretical model.

So far, organizational identification is introduced as the mediating variable and identification orientation as the moderating variable. According to the logical relationship, seven hypotheses are put forward to build a theoretical model to study the impact of workplace isolation on employees' negative behaviors. The research framework of this paper is as follows (Figure 1).

## 4. Method design and variable measurement

### 4.1. Sample extraction and investigation process

An electronic questionnaire was sent to the employees of enterprises in Beijing, Shanghai, Guangzhou, Shenzhen, Dongguan, Zhuhai and other regions. Before the survey, we fully communicated with the employees, informed that there was no right or wrong answer to all the respondents, and promised the anonymity and confidentiality of the questionnaire. At the same time, in order to avoid the deviation of the common method, the same group of people were required to fill in three parts of questionnaire at separated three time points. Each interval was 1 month, so the survey lasted for 3 months (from June to August 2021).

The first part of questionnaire (time point 1: early June 2021) involved the issue of workplace isolation. Five hundred questionnaires were distributed and 499 among those were recovered. The second part of questionnaire (time point 2: early July 2021) investigated employees' identification orientation and organizational identification. Four hundred ninety-nine questionnaires were issued and 415 among those were recovered. The third part of questionnaire (time point 3: early August 2021) investigated employees' work fatigue and turnover intention. Four hundred fifteen questionnaires were issued and 388 were recovered. In addition, common control variables are added, including gender, age, education level, wages, weekly working hours, etc.

After the completion of the survey, the three parts of questionnaires were matched according to the last four digits of the employee's mobile phone number, and the invalid questionnaires without answer were eliminated. Finally, 300 valid questionnaires were obtained from employees of enterprises in mainland China, and the effective recovery rate was 60.12%. In this questionnaire survey, there were 160 male employees, accounting for 53.3% of the total, and 140 female employees, accounting for the remaining 46.7%. Seventy-three unmarried employees, accounting for 24.3% of the questionnaire survey; and 227 married employees, accounting for remaining 75.7%. The average education level of the surveyed employees is junior college, the average age is 39.17 years, the average working period is 4.7 years, and the average weekly working hours is 50.45 h. Further details on the demographics of the group are shown in Table 1.

**Table 1:** Demographic characteristics of the sample.

**Variables**	**Number**	**Percentage**
**Gender**		
Male	160	53.3
Female	140	46.7
**Marital status**		
Married	227	75.7
Single	74	24.3
**Education**		
Secondary school	5	1.7
High school	65	21.7
University	200	66.7
Post-university	30	10
**Age**		
20–29	59	19.7
30–39	99	33
40–49	92	30.7
50–59	50	16.7
**Length of service (years)**		
1–5	198	66
6–10	101	33.7
>11	1	0.3
	**Mean**	**SD**
Working hours	50.5	6.9

### 4.2. Measuring tools

In order to ensure the reliability and validity of the questionnaire, the existing mature measurement was used for reference. Before the survey, according to the standard translation and back-translation procedure (43), the questionnaire was repeatedly proofread before issuance, and the measurement was finally accurately translated into Chinese. In addition, the Likert 5-point scale was used in the questionnaire (1–5 in the questionnaire respectively mean “Strongly disagree” to “Strongly agree”).

Workplace isolation: the questionnaire compiled by Marshall et al. (7) was used for the survey, with a total of 10 reverse questions. For example, “when I encounter problems, I have colleagues I can rely on”. The Cronbach's α coefficient is 0.908.

Organizational identification: the organizational identification scale compiled by Smidts et al. (34) was used, with five questions in total. For example, “I have a strong sense of belonging to the company I work for.” The Cronbach's α coefficient is 0.766.

Identification orientation: the individual identification orientation scale compiled by Johnson et al. (42) was used, with five questions in total. For example, “I am good at seizing the opportunity to prove my ability or talent is superior to others.” The Cronbach's α coefficient is 0.888.

Work fatigue: the work fatigue scale compiled by Frone and Tidwell (17) was used. The questionnaire can be divided into three dimensions, namely physical work fatigue, mental work fatigue and emotional work fatigue, with a total of 18 questions. For example, “Do you feel physically tired after work,” “Do you feel mentally tired after work,” “I feel very depressed after work.” The Cronbach's α coefficient is 0.956.

Turnover intention: the scale developed by Wayne et al. (44) was used with 5 questions. For example, “I am actively looking for another work,” “I am seriously considering leaving this firm.” The Cronbach's α coefficient is 0.874.

Control variables: because demographic variables also have an impact on work fatigue and turnover intention (17, 44), gender, age, education level, marital status, length of service, etc. are the main factors. At the same time, weekly working hours also have a differential impact on turnover intention (45). In order to verify the model more accurately, these factors were measured as control variables, including gender, age, education level, marital status, length of service and weekly working hours.

### 4.3. Data analysis methods

This study used SPSS 21.0 for Harman's one-way test, descriptive statistics, correlation analysis, and multiple regression analysis, and Mplus7.4 was used for confirmatory factor analysis. When testing the mediating effect, the confidence interval of the mediating effect was estimated by using the Bootstrap technique and the PROCESS program (46). When testing the moderated mediating effect, according to Edwards and Lambert (47) method and Bootstrap technology (Bootstrap), the significance of value and difference the indirect effect under the high and low moderators should be tested.

## 5. Results and analysis

### 5.1. Common method deviation test

The questionnaire was collected in a multi-stage manner suggested by Podsakoff et al. (48) to prevent possible common method deviations from the data. At the same time, Harman single-factor test was used to test whether this study was affected by the common method deviation. First, exploratory factor analysis without rotation was carried out on the questions. There were six common factors with eigenvalues >1. The variance interpretation rate of the first factor is 29.05%, which is less than the standard 40% (48). Through the comparison of a series of competition models, the analysis results are shown in the table below (Table 2).

**Table 2:** Results of confirmatory factor analysis (*n* = 300).

**Model**	***χ2***	***df***	****Δχ*2***	***RMSEA***	***SRMR***	***CFI***	***TLI***
Five-factor model (hypothesis)	1,563.36	850		0.053	0.055	0.904	0.901
Four-factor model (A+B)	1,815.87	854	252.51^***^	0.061	0.072	0.871	0.863
Four-factor model (B+C)	1,915.26	854	351.90^***^	0.064	0.086	0.857	0.849
Three-factor model (A+B+C)	2,422.03	857	858.67^***^	0.078	0.084	0.789	0.778
Two-factor model (A+B+C+D)	3,932.68	859	2,369.32^***^	0.109	0.147	0.586	0.565
Two-factor model (A+B+C+E)	2,998.23	859	1,434.87^***^	0.091	0.099	0.712	0.697
Single factor model (A+B+C+D+E)	4,529.46	860	2,966.10^***^	0.119	0.154	0.506	0.481

A-workplace isolation; B-organizational identification; C-identification orientation; D-work fatigue; E-turnover intention; “+” indicates integration.

It can be seen from above table that the fitting results of confirmatory factor analysis of single factor model(χ 2 = 4,529.46, df = 860, RMSEA = 0.119, SRMR = 0.154, CFI = 0.506, TLI = 0.481) is not ideal, indicating that there is no serious common method deviation between variables.

### 5.2. Confirmatory factor analysis

Since the variable data came from employee self-evaluation, it is necessary to use confirmatory factor analysis to test the discriminant validity of each variable (48). Therefore, the fitting indexes are selected to judge the fitting degree of the model. The Chi-square difference must reach a significant level, Root Mean Square Error of Approximation (RMSEA) must be <0.08, and the Comparative Fit Index (CFI) and Tucker-Lewis Index (TLI) must be > 0.9.

Table 2 shows the model adaptation of the five-factor model(χ2 = 1,563.36, df = 850, RMSEA = 0.053, SRMR = 0.055, CFI = 0.904, TLI = 901) is better than other competition models. All the adaptation indexes of the five-factor model have passed the test, which can determine the existence of discrimination of all variables. The five variables represent five different constructs.

### 5.3. Correlation analysis

In order to explore the relationship between workplace isolation, organizational identification, identification orientation, work fatigue and turnover intention, correlation analysis was conducted. The mean, standard deviation and correlation coefficient of key variables are shown in Table 3.

**Table 3:** Mean values, standard deviations and correlation coefficients of variables (*N* = 300).

**Variables**	**Mean**	**Standard deviation**	**1**	**2**	**3**	**4**	**5**	**6**	**7**	**8**	**9**	**10**
1 Gender	0.470	0.500										
2 Age	39.170	8.855	0.147^*^									
3 Years of education	14.930	1.964	−0.123^*^	−0.845^***^								
4 Marital status	0.760	0.430	0.110	0.724^***^	−0.527^***^							
5 Length of service	4.700	2.736	0.110	0.485^***^	−0.427^***^	0.424^***^						
6 Working hours	50.455	6.954	0.072	0.208^***^	−0.122^*^	0.298^***^	0.236^***^					
7 Workplace isolation	2.591	0.911	0.100	0.200^***^	−0.213^***^	0.134^*^	0.142^*^	0.035				
8 Organizational identification	3.601	0.805	−0.104	−0.045	0.053	0.002	−0.133^*^	0.124^*^	−0.395^***^			
9 Identification orientation	3.646	1.052	−0.075	−0.152^**^	0.220^***^	−0.006	−0.041	−0.024	−0.394^***^	0.239^***^		
10 Work fatigue	2.917	0.978	−0.030	−0.174^**^	0.185^**^	−0.310^**^	−0.046	−0.156^**^	0.218^***^	−0.317^***^	−0.206^***^	
11 Turnover intention	2.602	1.055	−0.011	−0.082	−0.004	−0.151^**^	−0.061	−0.110^*^	0.326^***^	−0.315^***^	−0.237^***^	0.311^***^

^***^p < 0.001, ^**^p < 0.01, ^*^p < 0.05.

Table 3 shows that the correlation coefficients between variables are significant, “workplace isolation” and work fatigue (r = 0.218, *p* < 0.001), “workplace isolation” and turnover intention (r = 0.326, *p* < 0.001) show a significant positive correlation, which provides preliminary evidence for subsequent hypotheses testing.

### 5.4. Hypothesis testing results

#### 5.4.1. Control variable inspection results

In order to clarify the impact of demographic variables, the gender, age, years of education, marital status, length of service and weekly working hours of employees are taken as control variables for regression analysis, and the results are shown in Table 4.

**Table 4:** Regression analysis model.

**Variables**	**Organizational identification**			**Work fatigue**			**Turnover intention**		

	**Model 1**	**Model 2**	**Model 3**	**Model 4**	**Model 5**	**Model 6**	**Model 7**	**Model 8**	**Model 9**
**Control variable**									
Gender	−0.100	−0.072	−0.073	−0.003	−0.023	−0.040	0.005	−0.020	−0.035
Age	−0.021	−0.005	0.017	0.339^*^	0.328^**^	0.327^**^	−0.142	−0.156	−0.157
Years of education	−0.004	−0.062	−0.062	0.284^**^	0.324^**^	0.309^**^	−0.206	−0.154	−0.168
Marital status	0.052	0.051	0.039	−0.426^***^	−0.425^***^	−0.413^***^	−0.135	−0.134	−0.123
Length of service	−0.174^**^	−0.153^*^	−0.162^**^	0.113	0.098	0.062	−0.009	−0.027	−0.061
Weekly working hours	0.161^**^	0.158^**^	0.164^**^	−0.091	−0.089	−0.052	−0.064	−0.061	−0.027
**Independent variable**									
Workplace isolation		−0.391^***^	−0.333^**^		0.270^***^	0.178^**^		0.350^***^	0.266^***^
**Mediating variable**									
Organizational identification						−0.237^***^			−0.216^***^
**Moderating variable**									
Identification orientation			0.067						
**Interaction terms**									
Workplace isolation ^*^ identification orientation			0.141^*^						
*R^2^*	0.054	0.199	0.226	0.134	0.204	0.249	0.040	0.156	0.194
*ΔR^2^*	0.054^*^	0.145^***^	0.172^***^	0.134^***^	0.069^***^	0.114^***^	0.040^*^	0.116^***^	0.153^***^

^***^p < 0.001, ^**^p < 0.01, ^*^p < 0.05.

According to model 1 in Table 4, employees' length of service and weekly working hours has an impact on Organizational Identification: the longer employees' length of service, the lower organizational identification (β = −0.174, *p* < 0.01); the longer the weekly working hours, the higher the employees' organization identification (β =0.161, *p* < 0.01).

According to model 4 in Table 4, employees' age, education level and marital status have an impact on work fatigue: older employees are more likely to feel fatigue at work (β = 0.339, *p* < 0.05); higher-educated employees are more likely to feel fatigue at work (β = 0.284, *p* < 0.01). Employees with high education are more likely to engage in complex work and the company have higher requirements for their ability, which leads to employees' higher likelihood to feel fatigue. Married employees are more likely to feel fatigue at work (β = −0.426, *p* < 0.001). Married employees put part of their limited energy in their families, they may suffer from work fatigue due to lack of energy when facing the work.

Therefore, it is necessary to fix the control variables when verifying the model. When processing the subsequent models, we analyzed the results in the case of controlling the employees' gender, age, education level, marital status, length of service, weekly working hours and other attributes.

#### 5.4.2. Main effect test results

In order to verify the impact of “workplace isolation” on work fatigue and turnover intention, regression analysis was conducted on work fatigue and turnover intention, respectively. According to model 5 in Table 4, “workplace isolation” has a significantly positive impact on work fatigue (β =0.270, *p* < 0.001), **Hypothesis 1 is confirmed**. According to model 8 in Table 4, “workplace isolation” has a significantly positive impact on turnover intention (β = 0.350, *p* < 0.001), **Hypothesis 2 is confirmed**.

#### 5.4.3. Mediating effect test results

In order to verify whether organizational identification plays a mediating role in the relationship between “workplace isolation” and work fatigue/turnover intention, according to model 6 in Table 4, organizational identification plays a partial mediating role in the relationship between “workplace isolation” and work fatigue (β = −0.237, *p* < 0.001). In order to further clarify this mediating effect, Process software was used to test based on Bootstrap method. The results are shown in Table 5.

**Table 5:** Bootstrap test for mediating effect 1.

**Mediating effect**	**Effect size**	**Standard error**	**95% Confidence interval**	
			**Lower confidence limit**	**Upper confidence limit**
Indirect effect	0.099	0.028	0.047	0.157
Direct effect	0.191	0.061	0.071	0.311

Bootstrap sample size *N* = 5,000.

It can be seen from Table 5 that the indirect and direct effects of organizational identification are significant, and 0 is not included in the 95% confidence interval, indicating that organizational identification plays a partial mediating role between “workplace isolation” and work fatigue. **Hypothesis 3 is confirmed**.

According to model 9 in Table 4, organizational identification plays a partial mediating role between “workplace isolation” and turnover intention (β = −0.216, *p* < 0.001). In order to further clarify this mediating effect, Process software was used to test based on Bootstrap method. The results are shown in Table 6.

**Table 6:** Bootstrap test for mediating effect 2.

**Mediating effect**	**Effect size**	**Standard error**	**95% Confidence interval**	
			**Lower confidence limit**	**Upper confidence limit**
Indirect effect	0.098	0.032	0.039	0.161
Direct effect	0.308	0.068	0.174	0.441

Bootstrap sample size *N* = 5,000.

It can be seen from Table 6 that the indirect and direct effects of organizational identification are significant, and 0 is not included in the 95% confidence interval, indicating that organizational identification plays a partial mediating role between “workplace isolation” and work fatigue. **Hypothesis 4 is confirmed**.

#### 5.4.4. Moderating effect test results

According to model 3 in Table 4, interaction term between employees' workplace isolation and individual identification orientation is significant (β = 0.141, *p* < 0.05). In addition, Δ*R*^2^ (the interaction term based on the control variables and the moderating variable) = 0.172 (*p* < 0.001), indicating that individual identification orientation plays a moderating role in the relationship between “workplace isolation” and organizational identification. In order to further clarify the moderating effect, Process software was used to test based on Bootstrap method. The results are shown in Table 7.

**Table 7:** Bootstrap test of moderating effect.

**Moderating effect**	**Effect size**	**Standard error**	**95% Confidence interval**	
			**Lower confidence limit**	**Lower confidence limit**
Low (+1SD)	−0.419	0.067	−0.550	−0.287
Middle	−0.194	0.051	−0.392	−0.194
High (+1SD)	−0.169	0.075	−0.317	−0.023

Bootstrap sample size *N* = 5,000.

**Figure 2:**
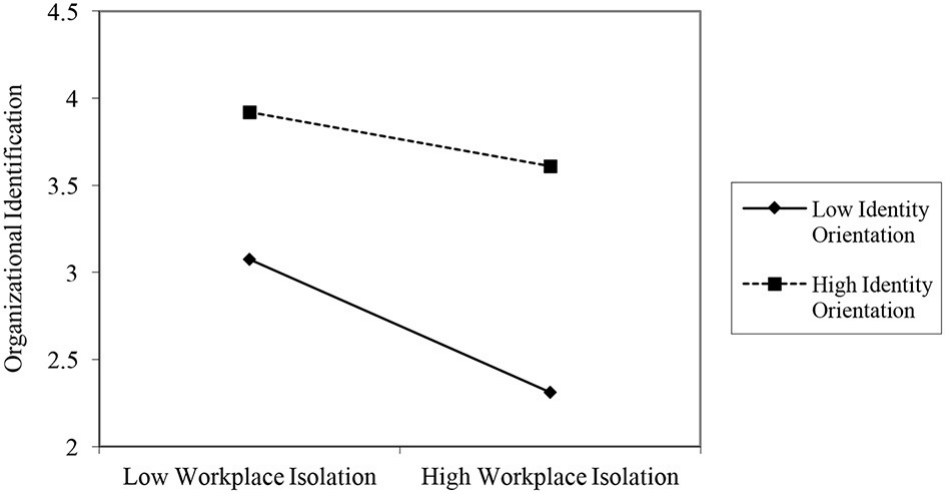
The impact of the interaction between “workplace isolation” and identification orientation on organizational identification.

It can be seen from Table 7 that the moderating effect of individual identification orientation is significant at both low and high levels, and 0 is not included in the 95% confidence interval. Then, by using method of Aiken et al. (49), the high and low levels of the moderating variables were adjusted (plus or minus) by one standard deviation (± 1SD) around the average, and the results are shown in Figure 2.

According to Figure 2, the higher the individual identification orientation, the weaker the positive impact of “workplace isolation” on organizational identification. **Hypothesis 5 is confirmed**.

#### 5.4.5. Moderated mediating effect test results

In order to test whether individual identification orientation moderates the indirect impact of “workplace isolation” on work fatigue through organizational identification, Bootstrap method is used to test the moderated mediating model. The analysis results are shown in Table 8.

**Table 8:** Moderated mediating effect Bootstrap test 1.

**Independent variable**	**Mediating variable**	**Moderating variable**	**Indirect effect**	**Standard error**	**95% Confidence interval**	
					**Lower confidence limit**	**Lower confidence limit**
		Low (-SD)	0.121	0.036	0.063	0.181
Workplace isolation	Organizational identification	Identification orientation				
		High (+SD)	0.049	0.028	0.006	0.096

Bootstrap sample size *N* = 5,000.

According to Table 8, under high degree of identification orientation, the indirect effect value is 0.049, and its 95% confidence interval is [0.006, 0.096]. Under low degree of identification orientation, the indirect effect value is 0.121, and its 95% confidence interval is [0.063, 0.181]. It can be seen that individual identification orientation also further negatively moderates the indirect impact of “workplace isolation” on work fatigue through organizational identification. **Hypothesis 6 is confirmed**.

Similarly, in order to test whether individual identification orientation moderates the indirect impact of “workplace isolation” on turnover intention through organizational identification, Bootstrap method is used to test the moderated mediating model. The analysis results are shown in Table 9.

**Table 9:** Moderated mediating effect Bootstrap test 2.

**Independent variable**	**Mediating variable**	**Moderating variable**	**Indirect effect**	**Standard error**	**95% Confidence interval**	
					**Lower confidence limit**	**Lower confidence limit**
		Low (-SD)	0.119	0.042	0.055	0.191
Workplace isolation	Organizational identification	Identification orientation				
		High (+SD)	0.048	0.028	0.006	0.097

Bootstrap sample size *N* = 5,000.

According to Table 9, under high degree of identification orientation, the indirect effect value is 0.048, and its 95% confidence interval is [0.006, 0.097]. Under low degree of identification orientation, the indirect effect value is 0.119, and its 95% confidence interval is [0.055, 0.191]. It can be seen that individual identification orientation further negatively moderates the indirect impact of “workplace isolation” on turnover intention through organizational identification. **Hypothesis 7 is confirmed**.

## 6. Conclusion and discussion

### 6.1. Conclusion

From the dual perspective of internal motivation of organizational environment and personal characteristics, this paper examines the mediating role of organizational identification between workplace isolation and work fatigue/turnover intention, and examines the moderating role of individual identification orientation, aiming to reveal the impact mechanism of workplace isolation on employees' negative work behaviors. The conclusions are as follows: Firstly, “workplace isolation” has a significantly positive impact on employees' work fatigue and turnover intention; Secondly, organizational identification plays a partial mediating role between “workplace isolation” and work fatigue/turnover intention; Thirdly, individual identification organizational identification, that is, the higher the individual identification orientation, it will inhibit more the negative impact of “workplace isolation” on organizational identification; Finally, individual identification orientation also further negatively moderates the indirect effect of “workplace isolation” on work fatigue and turnover intention through organizational identification, that is, compared with a lower degree of individual identification orientation, the positive effect of “workplace isolation” on work fatigue and turnover intention through organizational identification will be weakened for employees with higher identification orientation.

### 6.2. Theoretical contributions

First of all, this study verifies the impact of workplace isolation on employees' negative behaviors, which is conducive to a comprehensive and in-depth understanding of the negative consequences of workplace isolation, and provides new empirical evidence for enriching and expanding research on workplace isolation (4). The antecedent variables of work fatigue and turnover intention were expanded. This study fills the theoretical gap between workplace isolation and work fatigue and turnover intention, and enriches the cognition of workplace isolation and work negative behavior to a certain extent.

Second, organizational identification plays a mediating role in the process of workplace isolation 's impact on employees' negative work behaviors. Based on the social exchange theory, the research starts from the organizational environment of workplace isolation. It is difficult to meet the needs of belonging, self-esteem, control and existence of employees, reduce the identification with the organization, and show work fatigue and turnover tendency. At the same time, this study combines workplace isolation, organizational Identification, work fatigue, and turnover intention are integrated in a framework, revealing the pathway through which workplace isolation affects employees' negative work behaviors (7). Since previous studies on organizational identity paid less attention to interpersonal factors, the research results of this study on the relationship between workplace isolation and organizational identity also have certain enlightening significance for expanding the research on the antecedents of organizational identity.

Finally, this study verified that personal identity orientation negatively moderates the relationship between workplace isolation and organizational identity, and negatively moderates the mediating effect of organizational identification in the process of workplace isolation affecting employees' negative work behaviors. This conclusion supports the intrinsic motivation of self-identity orientation theory (50). Employees with high personal identity orientation can alleviate the negative impact of workplace isolation and reduce organizational identity with their inner motivation of independence and self-improvement. This paper expands the research on the boundary conditions of the relationship between workplace isolation and organizational identity, and reveals whether the internal motivation of employee identity orientation affects the path of workplace isolation on its negative work behavior through organizational identification.

### 6.3. Practical contributions

Accompanied by the attention of workplace isolation, alleviate its negative impact. This study provides practical guidance on how to deal with work-related negative behaviors brought about by workplace isolation. The study clarifies the impact mechanism of workplace isolation from the perspective of organizational isolation environment and internal self-improvement motivation, which can help organizations and employees clarify workplace isolation more accurately and objectively. Therefore, managers try to mitigate the negative impact of workplace isolation on individuals as much as possible. Organizations can try to create a mutual understanding, tolerance, and proactive organizational culture and communication mechanism, form a united, inclusive, and open working atmosphere, and prevent workplace isolation from happening.

***Strengthen employee identification with the organization*. **Since organizational identification plays a mediating role between workplace isolation and employees' negative work behaviors, companies can inhibit the positive impact of workplace isolation on employees' work fatigue and turnover intention by increasing employees' identification with the organization. Employees who identify with the organization, consider themselves a member of the organization, take the organization's goals as their work goals, and are more willing to make efforts and contributions (51). Therefore, managers actively communicate with employees to solve their work difficulties and enhance employees' sense of belonging to the organization (52). The human resources department can also appropriately increase team cohesion building activities to enhance employees' sense of identity.

***Improve employee identity orientation*. **Due to employees' different identification orientation, their feelings and behaviors toward “workplace isolation” are different. Therefore, managers should cultivate employees' identification orientation for the organization and improve employees' adaptability to negative events and emotions through targeted and planned training (53). In addition, when recruiting employees, the human resources department can test the employee's identification orientation and consider using the employee's identification orientation as a reference factor for hiring employees.

Finally, when facing decentralized office or home working, organizations can use information technology to strengthen interaction with employees (54), provide more remote communication platforms, establish channels for employee interaction, and provide personalized care for employees (55).

## 7. Limitation and future research directions

Admittedly, although this paper has made very valuable research conclusions, there are some research limitations need to be noted: due to the single source of data, the interpretation of causality in this paper is limited to a certain extent. It is suggested that longitudinal data should be adopted in subsequent studies in order to establish the causality more clearly; Secondly, due to the constraints of time, space and other factors, the sample size and data sources of this paper are not very extensive; Finally, this paper based the survey on employees themselves, the impact of family atmosphere on “workplace isolation” and the impact of organizational atmosphere and leadership were not considered. It is suggested that subsequent studies pay attention to these factors.

## Data availability statement

The raw data supporting the conclusions of this article will be made available by the authors, without undue reservation.

## Author contributions

Both authors listed have made a substantial, direct, and intellectual contribution to the work and approved it for publication.

## References

[1] Vaziri H Casper WJ Wayne JH Matthews RA. Changes to the work–family interface during the COVID-19 pandemic: examining predictors and implications using latent transition analysis. J Appl Psychol. (2020) 105:1073–87. 10.1037/apl0000819 32866024

[2] Pietromonaco PR Overall NC. Applying relationship science to evaluate how the COVID-19 pandemic may impact couples' relationships. Am Psychologist. (2021) 76:438–50. 10.1037/amp0000714 32700937

[3] Pietromonaco PR Overall NC. Implications of social Isolation, separation and loss during the COVID-19 pandemic for couples' relationships. Curr Opin Psychol. (2021) 43:189–94. 10.1016/j.copsyc.2021.07.014 34416682 PMC8881098

[4] Sahai S Ciby MA Kahwaji AT. Workplace isolation: a systematic review and synthesis. Int. J. Manag. (2020) 11:2745–58. 10.34218/IJM.11.12.2020.257

[5] Fox S Stallworth LE. Racial/ethnic bullying: exploring links between bullying and racism in the US workplace. J Vocat Behav. (2005) 66:438–56. 10.1016/j.jvb.2004.01.002

[6] Wu Z Liu J Hui C. Workplace ostracism and organizational citizenship behavior: the roles of organizational identification and collectivism. Nankai Business Rev. (2010) 13:36–44. 10.3969/j.issn.1008-3448.2010.03.006

[7] Marshall GW Michaels CE Mulki JP. Workplace isolation: exploring the construct and its measurement. Psychol Mark. (2007) 24:195–223. 10.1002/mar.20158

[8] Chandra G Robinson SL. They're talking about me again: the impact of being the target of gossip on emotional distress and withdrawal. In: Academy of Management Conference. Chicago. Paper presented at the annual meeting of the Academy of Management, Boston (2009).

[9] Yan Y Zhou E Long L Ji Y. The influence of workplace ostracism on counterproductive work behavior: the mediating effect of state self-control. Soc Behav Person Int J. (2014) 42:881–90. 10.2224/sbp.2014.42.6.881

[10] Neuman JH Baron RA. Workplace violence and workplace aggression: evidence concerning specific forms, potential causes, and preferred targets. J Manage. (1998) 24:391–419. 10.1177/014920639802400305

[11] Aquino K. Structural and individual determinants of workplace victimization: the effects of hierarchical status and conflict management style. J Manage. (2000) 26:171–93. 10.1177/014920630002600201

[12] Mulki JP Locander WB Marshall GW Harris EG Hensel J. Workplace isolation, salesperson commitment, and job performance. J Personal Sell Sales Manag. (2008) 28:67–78. 10.2753/PSS0885-3134280105

[13] Kwan HK Zhang X Liu J Lee C. Workplace ostracism and employee creativity: an integrative approach incorporating pragmatic and engagement roles. J Appl Psychol. (2018) 103:1358–66. 10.1037/apl0000320 29963897

[14] Jiang P Zhang L. Does conformity lead to gains? the effect of workplace ostracism on performance evaluation from a self-presentational view. Acta Psychologica Sinica. (2021) 53:400–12. 10.3724/SP.J.1041.2021.00400

[15] Deng X He S Lyu P Zhou X Ye Y Meng H. Spillover effects of workplace ostracism on employee family life: the role of need for affiliation and work-home segmentation preference. Acta Psychologica Sinica. (2021) 53:1146–60. 10.3724/SP.J.1041.2021.01146

[16] Wang D Qin Y Zhou W. The effects of leaders' prosocial orientation on employees' organizational citizenship behavior–the roles of affective commitment and workplace ostracism. Psychol Res Behav Manag. (2021) 14:1171–85. 10.2147/PRBM.S324081 34393526 PMC8354766

[17] Frone MR Tidwell MCO. The meaning and measurement of work fatigue: development and evaluation of the three-dimensional work fatigue inventory (3D-WFI). J Occup Health Psychol. (2015) 20:273. 10.1037/a0038700 25602275 PMC4505929

[18] Li L Müller R Liu B Wang Q Wu G Zhou S. Horizontal-leader identification in construction project teams in China: how Guanxi impacts coworkers' perceived justice and turnover intentions. Project Manag J. (2021) 52:577–91. 10.1177/87569728211042509

[19] Michele Kacmar K Andrews MC Van Rooy DL Chris Steilberg R Cerrone S. Sure everyone can be replaced… but at what cost? Turnover as a predictor of unit-level performance. Acad Manag J. (2006) 49:133–44. 10.5465/amj.2006.20785670

[20] Tajfel H Turner JC. Social psychology of intergroup relations. Annu Rev Psychol. (1982) 33:1–39. 10.1146/annurev.ps.33.020182.000245

[21] Tajfel H Turner JC. The social identity theory of intergroup behavior. In:Worchel S Austing WG, editors. The Psychology of Intergroup Behavior. Chicago: Nelson Hall (1986).

[22] Ghanbary A Haghshanas B Habibi E Abedi M. The investigation relationship between mental workload and occupational fatigue in the administrative staffs of a communications service company. Iranian J Health Safe Environ. (2019) 6:1221–5.

[23] Schneider B. The people make the place. Pers Psychol. (1987) 40:437–53. 10.1111/j.1744-6570.1987.tb00609.x

[24] Brewer MB Gardner W. Who is this “We”? levels of collective identity and self-representations. J Personal Soc Psychol. (1996) 71:83–93. 10.1037/0022-3514.71.1.83

[25] Johnson RE Chang CH Yang LQ. Commitment and motivation at work: the relevance of employee identity and regulatory focus. Acad Manag Rev. (2010) 35:226–45. 10.5465/AMR.2010.48463332

[26] Brickson S. The impact of identity orientation on individual and organizational outcomes in demographically diverse settings. Acad Manag Rev. (2000) 25:82–101. 10.5465/amr.2000.2791604

[27] Mann S Varey R Button W. An exploration of the emotional impact of tele-working via computer-mediated communication. J Manag Psychol. (2000) 15:668–90. 10.1108/0268394001037805431594466

[28] Myers D. The social psychology of sustainability. World Fut J Gen Evol. (2003) 59:201–11. 10.1080/02604020310133

[29] Winwood PC Winefield AH Dawson D Lushington K. Development and validation of a scale to measure work-related fatigue and recovery: the occupational fatigue exhaustion/recovery scale (OFER). J Occup Environ Med. (2005) 47:594–606. 10.1097/01.jom.0000161740.71049.c4 15951720

[30] Mobley WH. Intermediate linkages in the relationship between job satisfaction and employee turnover. J Appl Psychol. (1977) 62:237–40. 10.1037/0021-9010.62.2.237

[31] Cavanaugh MA Boswell WR Roehling MV Boudreau JW. An empirical examination of self-reported work stress among US managers. J Appl Psychol. (2000) 85:65–74. 10.1037/0021-9010.85.1.65 10740957

[32] Ying W Shasha L. Impacts of organizational silence on employee's negative behavior and its action mechanism. Sci Res Manag. (2017) 38:144–52.

[33] Poon JM. Distributive justice, procedural justice, affective commitment, and turnover intention: a mediation–moderation framework 1. J Appl Soc Psychol. (2012) 42:1505–32. 10.1111/j.1559-1816.2012.00910.x

[34] Smidts A Pruyn ATH Van Riel CB. The impact of employee communication and perceived external prestige on organizational identification. Acad Manag J. (2001) 44:1051–62. 10.2307/3069448

[35] Baumeister RF Leary MR. The need to belong: desire for interpersonal attachments as a fundamental human motivation. Psychol Bull. (1995) 117:497–529. 10.1037/0033-2909.117.3.497 7777651

[36] Leary MR Baumeister RF. The nature and function of self-esteem: sociometer theory. In: Advances in Experimental Social Psychology. Academic Press (2000). p. 1–62.

[37] Friedland N Keinan G Regev Y. Controlling the uncontrollable: effects of stress on illusory perceptions of controllability. J Pers Soc Psychol. (1992) 63:923–31. 10.1037/0022-3514.63.6.923 1460560

[38] Solomon S Greenberg J Pyszczynski T. A terror management theory of social behavior: the psychological functions of self-esteem and cultural worldviews. Adv Exp Soc Psychol. (1991) 24:93–159. 10.1016/S0065-2601(08)60328-712584055

[39] Dutton JE Dukerich JM Harquail CV. Organizational images and member identification. Admin Sci. (1994) Q39:239–63. 10.2307/2393235

[40] Ashforth BE Mael F. Social identity theory and the organization. Acad Manag Rev. (1989) 14:20–39. 10.5465/amr.1989.4278999

[41] Patchen M. (1970). Participation, Achievement and Involvement on the Job. Englewood Cliffs, NJ: Prentice Hall.

[42] Johnson RE Selenta C Lord RG. When organizational justice and the self-concept meet: consequences for the organization and its members. Organ Behav Hum Decis Process. (2006) 99:175–201. 10.1016/j.obhdp.2005.07.005

[43] Brislin RW. The wording and translation of research instruments. In:Lonner WJ Berry JW, editors. Field Methods in Cross-Cultural Research. Sage Publications, Inc (1986). p. 137–64.

[44] Wayne SJ Shore LM Liden RC. Perceived organizational support and leader-member exchange: a social exchange perspective. Acad Manag J. (1997) 40:82–111. 10.5465/257021

[45] Scott CR Connaughton SL Diaz-Saenz HR Maguire K Ramirez R Richardson B. The impacts of communication and multiple identifications on intent to leave: a multimethodological exploration. Manag Commun Quarterly. (1999) 12:400–35. 10.1177/0893318999123002

[46] Hayes AF. Introduction to Mediation, Moderation, and Conditional Process Analysis: A Regression-Based Approach. Guilford Publications, New York, NY: The Guilford Press (2017).

[47] Edwards JR Lambert LS. Methods for integrating moderation and mediation: a general analytical framework using moderated path analysis. Psychol Method. (2007) 12:1–22. 10.1037/1082-989X.12.1.1 17402809

[48] Podsakoff PM MacKenzie SB Lee JY Podsakoff NP. Common method biases in behavioral research: a critical review of the literature and recommended remedies. J Appl Psychol. (2003) 88:879–903. 10.1037/0021-9010.88.5.879 14516251

[49] Aiken LS West SG Reno RR. Multiple Regression: Testing and Interpreting Interactions. Newbury Park, NP: Sage (1991).

[50] Hornsey MJ. Social identity theory and self-categorization theory: a historical review. Soc Personal Psychol Compass. (2008) 2:204–22. 10.1111/j.1751-9004.2007.00066.x29999335

[51] Yue CA Men LR Ferguson MA. Examining the effects of internal communication and emotional culture on employees' organizational identification. Int J Bus Commun. (2021) 58:169–95. 10.1177/2329488420914066

[52] Salas-Vallina A Alegre J López-Cabrales Á. The challenge of increasing employees' well-being and performance: how human resource management practices and engaging leadership work together toward reaching this goal. Hum Resour Manage. (2021) 60:333–47. 10.1002/hrm.22021

[53] Brunetto Y Dick T Xerri M Cully A. Building capacity in the healthcare sector: a strengths-based approach for increasing employees' well-being and organisational resilience. J Manag Organ. (2020) 26:309–23. 10.1017/jmo.2019.53

[54] Saura JR Ribeiro-Soriano D Saldaña PZ. Exploring the challenges of remote work on Twitter users' sentiments: from digital technology development to a post-pandemic era. J Bus Res. (2022) 142:242–54. 10.1016/j.jbusres.2021.12.052

[55] Ewing M Men LR O'Neil J. Using social media to engage employees: Insights from internal communication managers. Int J Strat Commun. (2019) 13:110–32. 10.1080/1553118X.2019.157583032581299

